# False and True

**Published:** 1886-11

**Authors:** 


### FALSE AND TRUE.

The false deceives, the true exalts,—
Some things are true because they're false,
A glaring paradox;
The other evening at a fair,
A dainty maid with a merry air,
And a wealth of frizzled locks;
Whilst whirling, twirling like a top,
Half smothered by a nimble fop,
In a sort of a crazy waltz,
Was heard to cry in wild despair,
"My hair! my hair! I've lost my hair!"
*'T was true, because 't was false.*

